# Hormonal therapies and venous thrombosis: considerations for prevention and management—a reappraisal

**DOI:** 10.1016/j.rpth.2023.100155

**Published:** 2023-04-15

**Authors:** Jonathan Douxfils, Laure Morimont, Mitchell D. Creinin, Ulysse Gaspard, Jean-Michel Foidart

**Affiliations:** 1 Qualiblood sa, Namur, Belgium; 2 Department of Pharmacy, Faculty of Medicine, University of Namur, Namur Research Institute for Life Sciences, Clinical Pharmacology Research Group, Namur, Belgium; 3 Department of Obstetrics and Gynecology, University of California, Davis, Sacramento, California, USA; 4 Department of Obstetrics and Gynecology, University of Liège, Belgium; 5 Estetra SRL, An affiliate Company of Mithra Pharmaceuticals, Liège, Belgium; 6 University of Liège, Liège, Belgium

To the Editor,

We appreciate the response of LaVasseur et al. [1] to our Letter to the Editor on their article on the risk of venous thromboembolism (VTE) with hormonal therapies, in which they remark on a potential link of drospirenone (DRSP) with an increased risk of thromboembolism because of its antimineralocorticoid effect, which could interfere with the effect of aldosterone on hemostasis. These observations are debated in the literature [2] because aldosterone administration enhances thrombus burden, an effect that was prevented by cotreatment with eplerenone. Additionally, aldosterone treatment decreases bleeding time, induces platelet activation and degranulation, and increases the expression of plasminogen activator inhibitor-1, whereas antimineralocorticoids increase bleeding time, decrease platelet activation, and inhibit the expression of plasminogen activator inhibitor-1, fibrinogen, P-selectin, and interleukin-1β in a variety of rodent models [2]. Further, a recent retrospective cohort study reported that spironolactone treatment in adults with acne did not increase the risk of VTE (odds ratio, 0.57; 95% CI, 0.31-1.06) or pulmonary embolism (odds ratio, 0.60; 95% CI, 0.26-1.37) compared with that in patients being treated with tetracycline antibiotics [3]. DRSP, as a progestin-only contraceptive at a dose of 4 mg, has not been categorized by regulatory bodies as having a different risk of VTE from other progestin-only contraceptives. In addition, hemostatic investigations of individual coagulation factors did not reveal the clinically relevant impact of this DRSP at 4 mg (Figure A) [4]. Interestingly, estetrol (E4), in combination with DRSP, has a coagulation profile similar to DRSP alone, whereas a clear difference is observed with the ethinylestradiol (EE)/DRSP combination.

**Figure:**
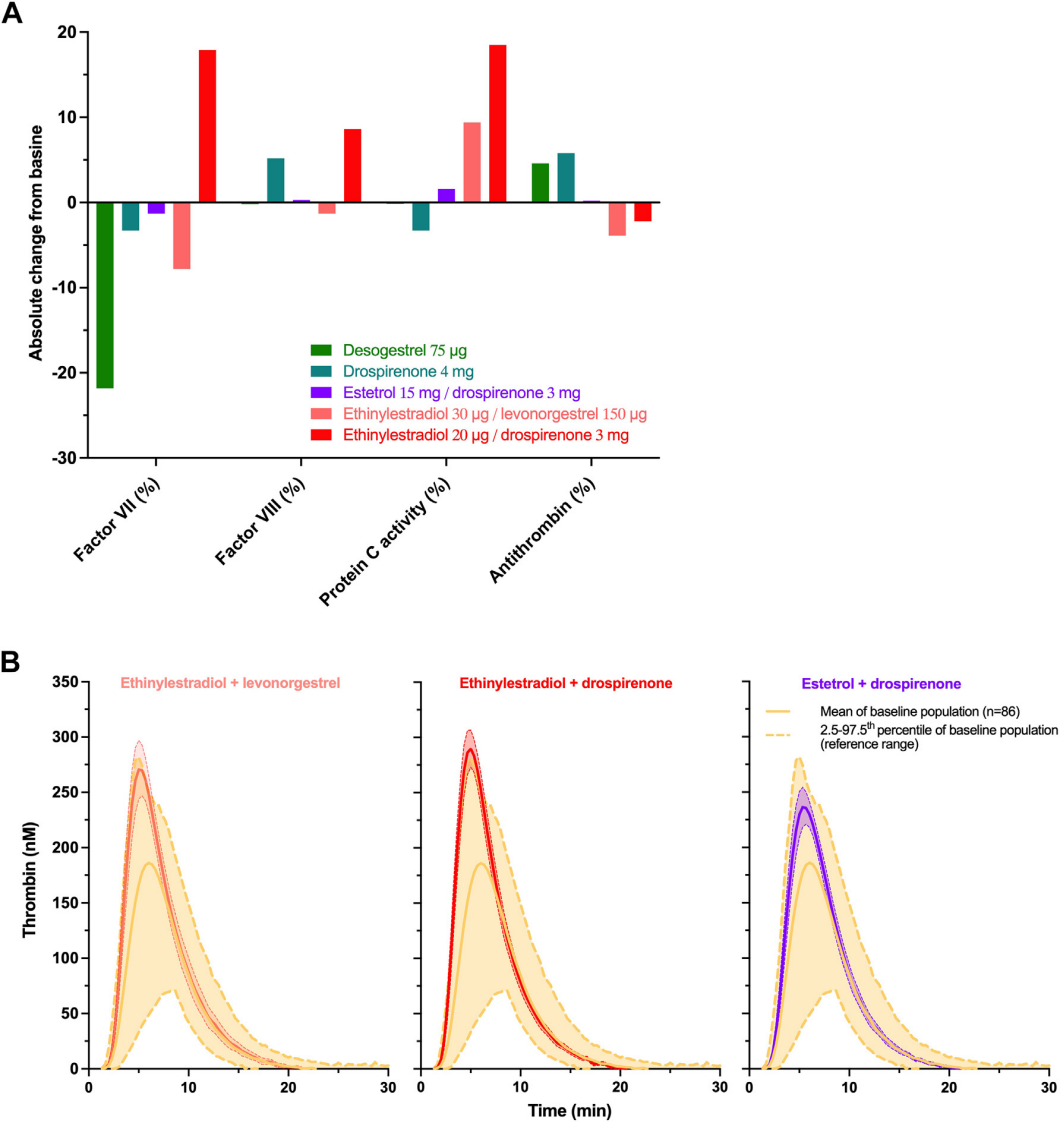
(A) A comparison of the absolute change from baseline in a common series of coagulation factors between 75-μg desogestrel and 4-mg drospirenone; 2 progestin-only contraceptives; and the combined oral contraceptives (COCs) 15-mg estetrol (E4) + 3-mg drospirenone, 20-μg ethinylestradiol **+** 3-mg drospirenone, and 30-μg ethinylestradiol **+** 150-μg levonorgestrel. Different profiles were observed for products containing ethinylestradiol, whereas progestin-only contraceptives and E4-containing pills are either neutral or have decreased hypercoagulability. (B) Comparison of thrombin generation profiles of ethinylestradiol-containing COCs versus those of E4-containing COCs. Products that contain ethinylestradiol showed mean thrombograms outside the reference range, whereas E4 does not clinically impact thrombin generation. COC, combined oral contraceptive; EE, ethinylestradiol.

Evidence is also accumulating regarding the correlation between coagulation markers, such as thrombin generation-based assays, and the risk of VTE observed in phase 4 studies. Haverinen et al. [5] also reported that thrombin generation was lower after exposure to estradiol valerate/dienogest compared with EE/dienogest, the latter being associated with a higher risk of VTE compared with EE/levonorgestrel (adjusted hazard ratio, 1.57; 95% CI, 1.07-2.30) [6], whereas the former has a similar lower risk of VTE (adjusted hazard ratio, 0.40; 95% CI, 0.20-1.10) [7]. We also observed that EE-containing combined oral contraceptives (COCs) have higher thrombin generation than E4/DRSP and that the magnitude of the impact of EE-containing COCs can be considered clinically relevant (Figure B).

Lastly, the plasma coagulation inhibitor subcommittee of the ISTH SSC has recently recommended the use of the endogenous thrombin potential-based activated protein C (APC) resistance assay to evaluate hormone-induced acquired APC resistance, an independent risk factor for VTE [8]. The model presented in our initial response is in line with this expert recommendation because we showed that acquired APC resistance is correlated with the risk of VTE with different COC preparations. This validates our observation that E4/DRSP leads to lower acquired APC resistance compared with EE-containing COC, reducing the burden of this independent risk factor [9].

Therefore, we reiterate our conclusion that the choice of estrogen matters when considering the risk of VTE and that classification of COC should be reappraised considering the latest scientific evidence.
